# Associations of Diabetes-Related Distress, Disordered-Eating Risk, and Self-Perceived Health with Glycemic Outcomes in Adolescents with Type 1 Diabetes: An Exploratory Cross-Sectional Study

**DOI:** 10.3390/nu18172916

**Published:** 2026-09-05

**Authors:** Marta García-Poblet, Isabel Sospedra, Brisa Florencia Formilan, Esther Soler-Climent, Manuel Antonio Alberola-Chazarra, José Miguel Martínez-Sanz

**Affiliations:** 1 Nursing Department, Faculty of Health Science, University of Alicante, 03692 Alicante, Spain; marta.poblet@ua.es (M.G.-P.); florformilan@gmail.com (B.F.F.); josemiguel.ms@ua.es (J.M.M.-S.); 2 Research Group on Applied Dietetics, Nutrition and Body Composition (DANuC), University of Alicante, 03692 Alicante, Spain; 3 Research and Innovation Area, Health Department of General Hospital of Elche, 03203 Elche, Alicante, Spain; soler_estcli@gva.es; 4 Traumatology and Neurosurgery Service, General Hospital of Elche, 03203 Elche, Alicante, Spain; 5 Pediatrics Unit, General Hospital of Elche, 03203 Elche, Alicante, Spain; alberola_man@gva.es

**Keywords:** adolescents, eating disorders, metabolic control, psychological distress, self-perceived health, type 1 diabetes mellitus

## Abstract

**Background/Objectives:** Adolescents with type 1 diabetes mellitus (T1DM) are particularly vulnerable to psychological distress, disordered-eating (DE) risk and negative self-perceived health status (SPHS), which may compromise metabolic control and self-care. Although these factors have been studied individually, the usefulness of brief and clinically feasible screening tools to detect these dimensions remains underexplored. This study aimed to: (1) examine associations between distress, DE risk and SPHS; (2) explore their relationships with glycemic control, clinical profile and self-care; and (3) assess the influence of age and diabetes duration on these outcomes. **Methods:** A cross-sectional study was conducted in 37 adolescents with T1DM in Spain. Sociodemographic, anthropometric and clinical variables were obtained from medical records. Distress, DE risk and self-care were assessed using validated questionnaires, while SPHS was measured using single-item question. Pearson and Spearman correlations were performed. **Results:** Distress was present in 48.6% of participants and DE risk in 29.7%, while 67.6% reported positive SPHS. Distress correlated with higher HbA1c and lower TIR. DE risk correlated with BMI. Lower SPHS was associated with higher HbA1c. Age and diabetes duration were associated with greater glycemic variability, and diabetes duration also correlated with higher glucose and TyG-based insulin resistance indexes. No significant associations were found for self-care behaviors. **Conclusions:** Distress, DE risk and SPHS, assessed through feasible screening tools, showed meaningful associations with metabolic outcomes in adolescents with T1DM. Integrating brief psychological screening tools into routine care may facilitate early identification of psychological vulnerability profiles in this population during routine practice.

## 1. Introduction

Type 1 diabetes mellitus (T1DM) is a metabolic disorder characterized by autoimmune destruction of pancreatic ß-cells, resulting in insulin deficiency [1]. Insulin is essential for blood glucose regulation, and its absence is associated with increased mortality [1]. In 2021, T1DM affected an estimated 8.4 million people worldwide, including 1.5 million under 20 years of age [2].

Adolescence, defined as the period between 10 and 19 years of age, is characterized by rapid physical, psychological and social development, as well as growing autonomy, decision-making skills and emotional fluctuations [3]. These characteristics, together with behavioral instability and rebellion, may compromise treatment adherence and glycemic control in adolescents with T1DM [4,5].

Diabetes-related distress is common among individuals with T1DM, negatively influencing adherence and mental health [5,6]. It is associated with numerous psychological disorders, one of the most common being eating disorders [5,7,8]. Currently, adolescents with T1DM show a twofold higher prevalence of eating disorders compared to their peers without diabetes [9], to the extent that a specific eating disorder has been identified in this population [8,9]. Psychological distress in adolescents with T1DM has also been associated with poorer glycemic outcomes, reduced adherence and increased risk of diabetes-related complications [6,10], which points out its clinical relevance in pediatric diabetes care.

Several factors may contribute to psychological distress and disordered-eating (DE) risk in adolescents with T1DM. Excess adiposity (normally assessed through body mass index (BMI), although in pediatric populations, it is more appropriate to use BMI-for-age percentiles or z-scores [11]) is especially relevant, since higher BMI and body dissatisfaction have been linked to increased vulnerability to DE [12]. DE in adolescents with T1DM has also been associated with greater glycemic variability, increased risk of acute complications such as severe hypoglycemia or diabetic ketoacidosis, and reduced diabetes-related quality of life, further pointing out its clinical relevance in this population [12,13,14]. Additionally, self-care behaviors such as dietary habits, physical activity or glycemic monitoring through continuous glucose monitoring (CGM) also affect self-perceived health status (SPHS) and DE risk [12,15]. SPHS reflects the individual’s subjective evaluation of their physical and emotional health and has been linked to treatment adherence in adolescents with T1DM, since negative SPHS has been associated with higher distress and maladaptive behaviors [16,17].

Metabolic control is fundamental for preventing both short- and long-term outcomes [18,19] and depends on glycemic control, anthropometric and biochemical parameters, and adherence to self-care practices [18]. Psychological distress, DE risk and SPHS may impair metabolic control directly or indirectly, particularly during adolescence, when disease management is often inconsistent [5]. Moreover, age and diabetes duration are also relevant, since older adolescents tend to exhibit lower adherence due to increased independence or psychological mechanisms such as denial [20,21], while longer diabetes duration is associated with psychological fatigue and metabolic deterioration [4,22]. In addition to traditional biochemical parameters, emerging metabolic markers such as the triglyceride–glucose (TyG) index have shown evidence of utility in adults with and without diabetes [23,24,25,26], and in adolescents with cardiometabolic risk [27,28,29]. Recent studies suggest that TyG-based indices may also be promising for characterizing metabolic status in adolescents with T1DM [30].

Although some studies have investigated the relationship between psychological distress, DE risk, anthropometrics and treatment adherence in individuals with T1DM, most have focused on adults [31,32,33]. Evidence in adolescents remains limited and existing research rarely evaluates these three psychological dimensions together, despite the availability of brief, clinically feasible screening tools. Therefore, this study aimed to (1) examine associations between psychological distress, DE risk and SPHS in adolescents with T1DM; (2) explore their relationships with glycemic control, anthropometry, biochemical profile and self-care behaviors; and (3) explore the association of age and diabetes duration with these outcomes. We hypothesize that psychological distress, DE risk and SPHS are associated with poorer metabolic control in adolescents with T1DM, and that age and diabetes duration may modulate these associations.

## 2. Materials and Methods

### 2.1. Design

This cross-sectional study was conducted at the pediatric unit of the General Hospital of Elche (Alicante, Spain). The study design and manuscript development followed the STROBE-nut guidelines for nutritional epidemiology, an extension of the STROBE recommendations for observational research [34,35].

### 2.2. Population

This was a feasibility, single-center study including all adolescents with T1DM attending the pediatric unit during the recruitment period. Adolescents aged 10–19 years with confirmed T1DM, with or without a CGM device, were recruited through non-probabilistic convenience sampling during routine outpatient visits. Exclusion criteria were hospitalization and pregnancy. During this period, 39 adolescents had scheduled routine visits, although 2 of them did not attend their appointment and could not be approached. All 37 adolescents who attended were eligible, agreed to participate, and were included in the final analyses. A preliminary sample size estimation was performed to obtain an approximate minimum number of participants required for correlational analyses, which indicated that around 30 adolescents would be needed. Consequently, the sample size reflected the number of eligible patients, and the study was conceived as exploratory. Therefore, the sample size was not determined by a formal power requirement. With the final sample of 37 participants, the smallest correlation detectable with 80% power at α = 0.05 was approximately r = 0.42.

### 2.3. Data Collection

Two trained healthcare professionals collected data in person between May and December 2024 during routine consultations, extracting sociodemographic and clinical variables from electronic health records, biochemical parameters from the latest blood tests and additional variables from validated self-administered questionnaires (all administered in their validated Spanish versions) completed by participants. To minimize external influences on adolescents’ emotional responses during data collection, all questionnaires were administered in a standardized clinical environment. Although legal guardians were present in the consultation area, adolescents completed the questionnaires individually at a separate table, with sufficient space, privacy, and time to respond. Trained healthcare professionals provided neutral instructions, clarified that there were no right or wrong answers, and ensured that all items were completed. Participants were informed that responses were anonymous and confidential, in accordance with the ethics protocol. The dataset (Microsoft Excel 365) generated in this study has been deposited in Zenodo under restricted access (DOI: https://doi.org/10.5281/zenodo.17573334). Data are available from the corresponding author upon reasonable request.

### 2.4. Variables

#### 2.4.1. Outcome Variables: Psychological Distress, EDE Risk and SPHS

Psychological distress was assessed using the 2-item Diabetes Distress Scale short form (DDS2S) [36], which evaluates diabetes-related emotional burden. Each item is scored on a 6-point Likert scale (from 1 = “not a problem” to 6 = “a very serious problem”), and the final score is the mean of both items. Higher scores indicate greater diabetes-related distress, with a mean ≥3 commonly used as a threshold for clinically relevant psychological distress. Although the DDS2S has been translated into Spanish and previously used in Spanish research [37,38], no formal psychometric validation exists for Spanish adolescents with type 1 diabetes; therefore, its use in this study should be considered exploratory.

DE risk was assessed with the EPAD-R, the Spanish-validated version of the original English DEPS-R (Diabetes Eating Problem Survey–Revised) [39]. The EPAD-R consists of 16 items evaluating thoughts, attitudes, and behaviors related to eating, weight control, and insulin manipulation in adolescents with T1DM. Each item is scored on a 5-point Likert scale (0 = “never” to 4 = “always”), and the total score is obtained by summing all item responses, with higher scores indicating greater DE risk. A total score ≥20 was used as the cut-off for defining DE risk. This is a screening tool, not a diagnostic instrument [40].

SPHS was rated on a 5-point scale (1 = “very unwell” to 5 = “extremely well”) in response to “How healthy do you believe you are?”. SPHS was measured using a single-item format commonly used in adolescent research and previously applied in studies of adolescents with T1DM [16,17]. Single-item measures of perceived health are widely used as global indicators of subjective health status and offer a practical approach for capturing overall health perception in clinical and research settings [41]. All questionnaires were completed during the clinical visit.

#### 2.4.2. Predictive Variables

All measurements were obtained at the closest feasible time point to the clinical visit, according to the practical and clinical requirements of each variable.

**Sociodemographic and anthropometric variables.**

Sex was defined as biological sex (female or male) as recorded in clinical records. Body mass and stature were measured using a calibrated scale and stadiometer (SECA 704s, Hamburg, Germany) following the ISAK standard [42]. BMI, BMI-for-age percentile and z-score were calculated using WHO AnthroPlus software (version 1.0.4) [43]. Adult BMI categories were not used for clinical interpretation; BMI was analyzed as a continuous variable, and weight status was classified using BMI-for-age percentiles (underweight (<5th), normal (5th–<85th), overweight (85th–<95th) and obesity (≥95th) and BMI-for-age z-scores (underweight (<−2), normal (−2 to 0.99), overweight (1–1.99) and obesity (> 2) [11]. From medical records, age at the time of the visit was also collected, and diabetes duration was derived from age at diagnosis.

**Glycemic control variables.**

Coefficient of variation (CV) and time in range (TIR) were obtained from CGM device records as mean values over the previous three months preceding the clinical visit, which aligns with the physiological period reflected by HbA1c [19]. Normal values were defined as CV ≤ 36% [44] and TIR ≥ 70% [19]. HbA1c was measured during clinical visits using the Alere Afinion AS100 Analyzer (Abbott, IL, USA) and categorized using a ≤6.5% threshold, in accordance with the most recent ISPAD recommendations for adolescents using advanced diabetes technologies such as CGM devices [19] and consistent with the latest ADA update for children and adolescents [13].

**Biochemical variables.**

Biochemical variables, including glucose, total cholesterol, LDL cholesterol, HDL cholesterol and triglycerides, were obtained from routine blood samples. These laboratory tests corresponded to the routine blood work performed within the three months preceding the clinical visit, which is the standard interval between pediatric diabetes follow-up visits in our clinical setting, and were ordered specifically for review during that encounter. All parameters were obtained under fasting conditions and from the same fasting blood sample. From these, triglyceride–glucose (TyG) index and TyG-BMI were calculated as markers of insulin resistance [45]. TyG index was calculated using the standard formula ln [triglycerides (mg/dL) × glucose (mg/dL)/2], and TyG-BMI index was calculated as TyG × BMI [45].

**Self-care variables.**

Self-care behaviors included adherence to the Mediterranean diet, physical activity and active CGM time. Diet adherence was assessed using the KIDMED questionnaire [46], composed of 16 items (12 items scored: +1, and 4 items reverse-scored: −1), yielding a total score from −4 to 12 and categorized as low (≤3), moderate (4–7), or high adherence (≥8). Physical activity was evaluated using the Spanish-validated Adolescent Physical Literacy Questionnaire (APALQ) [47], consisting of five Likert-type items assessing weekly activity frequency (1 = never to 5 = ≥4 times/week). Total scores ranged from 5 to 25 and were classified as sedentary (5–10), moderately active (11–16), or highly active (>16). Both the KIDMED and APALQ questionnaires were completed during the clinical visit. Active CGM time was obtained from device records, calculated over the three months preceding the clinical visit, with adequate usage defined as ≥70% of wear time [48].

### 2.5. Ethics

The study adhered to the Declaration of Helsinki and was approved on 30 April 2024 by the Research Ethics Committee of the General Hospital of Elche (PI 58/2024). Participants and their legal guardians were informed of the objectives of the study and provided informed consent.

### 2.6. Statistical Analysis

Data were analyzed using SPSS version 28.0 (IBM Corp., Armonk, NY, USA). Descriptive analysis included percentages, mean and standard deviations (SDs). Trends across categories were explored using cross-tabulations. Correlations were assessed with Pearson or Spearman tests, depending on normality assessed using the Shapiro–Wilk test. Outliers were inspected visually through scatterplots, and no extreme values required removal. Linearity was assessed through scatterplots and linear fit lines before selecting Pearson or Spearman correlation. Ordinal variables were analyzed using Spearman’s rho. Missing data were handled using pairwise deletion, as recommended for correlation matrices. Statistical significance was set at *p* < 0.05. For statistically significant correlations, 95% confidence intervals were calculated. In addition, *p*-values from all correlation analyses were adjusted for multiple comparisons using the Benjamini–Hochberg false-discovery-rate procedure. Adjusted and unadjusted *p*-values are reported for all correlations.

Multiple linear regression models were fitted using age, sex, and diabetes duration as covariates. Each psychological variable that showed a significant bivariate correlation with a clinical outcome was entered as the single predictor in its corresponding model, following a limited-adjustment strategy to avoid overfitting. Model assumptions were verified, and collinearity was assessed using tolerance and variance inflation factor (VIF) values.

Internal consistency was assessed using Cronbach’s alpha for all questionnaires with more than two items (KIDMED, EPAD-R and APALQ). For the DDS2S, which consists of only two items, internal consistency was evaluated using the Pearson correlation between both items.

## 3. Results

### 3.1. Sample Characteristics

Table 1 summarizes sociodemographic, psychological and glycemic data for 37 participants aged 10–19 years (40.6% female). CGM devices were used by 97.3% of the sample, with 91.6% active more than 70% of the time. Mean diabetes duration was 7.2 ± 4 years. Mean CV was 43.4 ± 11.7 and 83.3% of participants showed CV above the recommended threshold. Similarly, mean TIR was 58.8 ± 14 with 75% of participants not reaching the target. However, HbA1c indicated a lower percentage (75.6%) of participants not achieving optimal glycemic control (mean 7 ± 0.9). No participant had a previously diagnosed eating disorder and all used the same CGM device model (FreeStyle Libre 2; Abbott Diabetes Care, Alameda, CA, USA).

Psychological measures showed 48.6% screened positive for psychological distress and 91.1% screened positive for DE risk. SPHS was “neutral” in 32.4% of participants, “good well-being” in 59.5% and “optimal” in 8.1%. KIDMED indicated 16.2% had a low-quality diet, 59.5% had an improbable diet and 24.4% had an optimal diet. APALQ classified 18.9% as sedentary, 45.9% as moderately active and 35.2% as active. Internal consistency analyses conducted in our sample indicated adequate reliability levels for the instruments used: α = 0.613 for the EPAD-R and α = 0.550 for the APALQ. As expected for heterogeneous indices, the KIDMED showed lower internal consistency (α = 0.359). For the DDS2S, the correlation between its two items was significant (r = 0.357; *p* = 0.030), supporting adequate internal consistency for a brief two-item measure.

Table 2 shows anthropometric and biochemical data. Based on BMI, 72.9% were normal weight, with percentile and z-score classifying 71.4% as normal weight. Overweight and obesity rates were 13.5% and 2.8% by BMI, versus 17.2% and 11.4% by percentile and z-score. Mean serum glucose was 153.6 mg/dL, total cholesterol 167.8 mg/dL, and triglycerides 67.6 mg/dL. Mean TyG index was 8.4 and TyG-BMI 187.2.

Descriptive analysis conducted through cross-tabulations suggested exploratory patterns between psychological distress and several clinical categories, although these distributions were not fully consistent across variables. Psychological distress was slightly more frequent among participants with overweight or obesity (based on z-score and percentile), suboptimal glycemic control (higher HbA1c and CV) and elevated total cholesterol. Conversely, participants with normal weight, controlled HbA1c and lower glycemic variability more often showed no distress. These exploratory patterns (Supplementary Tables S1–S5) should be interpreted cautiously due to limited cell sizes. Cross-tabulations for DE risk categories were explored, but small cell sizes precluded meaningful interpretation. All patterns were further examined in correlational analyses (Table 3 and Table 4).

**Table 1:** Sociodemographic, clinical, glycemic, psychological, and self-care characteristics of adolescents with type 1 diabetes mellitus.

	n (%)	Mean ± SD
**Gender**	37 (100%)	
Male	22 (59.4%)	
Female	15 (40.6%)	
**Age**	37 (100%)	14.0 ± 2.6
10 to 14 years old	18 (48.6%)	12.7 ± 1.3
15 to 19 years old	19 (51.4%)	16.9 ± 1.7
**Diabetes duration**	37 (100%)	7.2 ± 4.2
≤5 years	16 (43.2%)	3.5 ± 1.3
>5 years	21 (56.8%)	10.0 ± 3.4
**Use of CGM device**	37 (100%)	
Yes	36 (97.3%)	
No	1 (2.7%)	
**Active CGM time**	36 (97.3%)	89.3 ± 10.4
≥70% of time	33 (91.6%)	91.7 ± 6.8
<70% of time	3 (8.4%)	63.0 ± 3.4
**CV**	36 (97.3%)	43.4 ± 11.7
C36% (controlled)	6 (16.7%)	33.0 ± 2.6
>36% (not controlled)	30 (83.3%)	45.4 ± 11
**TIR**	36 (97.3%)	58.8 ± 14
**≥70% (controlled)**	9 (25%)	77.2 ± 6.4
**<70% (not controlled)**	27 (75%)	52.7 ± 9.8
**HbA1c**	37 (100%)	7.0 ± 0.9
≤6.5% (controlled)	9 (24.4%)	5.9 ± 0.4
>6.5% (not controlled)	28 (75.6%)	7.4 ± 0.7
**DDS2S**	37 (100%)	2.7 ± 0.9
≥3 points (presence of distress)	18 (48.6%)	3.5 ± 0.5
<3 points (absence of distress)	19 (51.4%)	1.9 ± 0.5
**EPAD-R**	37 (100%)	27.2 ± 6.1
≥20 points (positive screening for DE risk)	34 (91.9%)	28 ± 5.7
<20 points (negative screening for DE risk)	3 (8.1%)	18.3 ± 1.1
**SPHS**	37 (100%)	3.7 ± 0.6
1 (worst well-being)	0 (0%)	
2 (moderate well-being)	0 (0%)	
3 (neutral)	12 (32.4%)	
4 (good well-being)	22 (59.5%)	
5 (optimal well-being)	3 (8.1%)	
**KIDMED**	37 (100%)	6.0 ± 2.1
Low-quality diet	6 (16.2%)	2.5 ± 0.8
Improvable diet	22 (59.4%)	6.0 ± 0.9
Optimal diet	9 (24.4%)	8.5 ± 0.7
**APALQ**	37 (100%)	14 ± 3.7
Sedentary	7 (18.9%)	8.3 ± 0.9
Moderately active	17 (45.9%)	13.8 ± 1.4
Active	13 (35.2%)	18.0 ± 1.2

CGM: continuous glucose monitor; CV: coefficient of variation; TIR: time in range; HbA1c: glycated hemoglobin; DDS2S: Spanish translation of the two-item Diabetes Distress Scale; EPAD-R: Encuesta de Problemas Alimentarios en Diabetes–Revisada (the Spanish version of the revised DEPS-R); DE: disordered-eating; SPHS: self-perceived health status; KIDMED: Mediterranean Diet Quality Index for children and adolescents; APALQ: Adolescent Physical Literacy Questionnaire.

**Table 2:** Anthropometric and biochemical characteristics of adolescents with type 1 diabetes mellitus.

	n (%)	Mean ± SD
**BMI**	37 (100%)	21.5 ± 3.5
**Percentile (BMI for age)**	35 (94.5%)	61.5 ± 26
<P5 (underweight)	0 (0%)	
P5 to P84.9 (normal weight)	25 (71.4%)	51.6 ± 21.7
P85 to P94.9 (overweight)	6 (17.2%)	90.7 ± 3.5
≥P95 (obesity)	4 (11.4%)	99.2 ± 0.2
**Z-score (BMI for age)**	35 (94.5%)	0.5 ± 1
<−2.00 (underweight)	0 (0%)	
−2.00 to +0.99 (normal weight)	25 (71.4%)	−0.01 ± 0.5
+1.00 to 1.99 (overweight)	6 (17.2%)	1.3 ± 0.2
≥2.00 (obesity)	4 (11.4%)	2.7 ± 0.5
**Glucose (mg/dL)**	30 (81%)	153.6 ± 51.4
**Total cholesterol (mg/dL)**	28 (75.6%)	167.8 ± 40.7
**LDL cholesterol (mg/dL)**	24 (85.7%)	96.1 ± 25.7
**HDL cholesterol (mg/dL)**	24 (85.7%)	65.1 ± 13.9
**Triglycerides (mg/dL)**	30 (81%)	67.6 ± 26.8
**TyG index**	30 (81%)	8.4 ± 0.5
**TyG-BMI index**	30 (81%)	187.2 ± 36

BMI: body mass index; TyG index: triglyceride-glucose index.

### 3.2. Correlation Analysis

Table 3 shows the adjusted and unadjusted correlations between psychological variables (distress, DE risk and SPHS) and clinical, anthropometric, biochemical and self-care variables. Psychological distress correlated positively with HbA1c (r = 0.401, *p* = 0.014) and negatively with TIR (r = –0.355, *p* = 0.034). DE risk showed a significant correlation with BMI (r = 0.333, *p* = 0.044). SPHS correlated negatively with HbA1c (r = –0.330, *p* = 0.046). However, none of these associations remained significant after adjusting for multiple comparisons.

Table 4 presents the adjusted and unadjusted correlations of age and diabetes duration with clinical, anthropometric, biochemical and self-care variables. Age correlated positively with CV (r = 0.354, *p* = 0.034) and BMI (r = 0.340, *p* = 0.039), while diabetes duration also correlated positively with CV (r = 0.347, *p* = 0.038), BMI (r = 0.472, *p* = 0.003), serum glucose (r = 0.474, *p* = 0.011), TyG index (r = 0.475, *p* = 0.011) and TyG-BMI index (r = 0.492, *p* = 0.007). After adjusting for multiple comparisons, only the associations between diabetes duration and BMI, serum glucose, TyG index and TyG-BMI index remained statistically significant (all adjusted *p* = 0.044). The principal bivariate associations represented in Table 3 and Table 4 are illustrated in Supplementary Figures S1–S5.

Table 5 summarizes the multiple linear regression models examining psychological variables as predictors of the clinical outcomes that showed significant bivariate associations (TIR, HbA1c and BMI), adjusting for age, sex and diabetes duration. In model 1 (TIR), psychological distress did not reach statistical significance (B = −4.841, *p* = 0.053), although the association showed a trend toward lower TIR with higher distress. In model 2A (HbA1c), psychological distress remained a significant predictor (B = 0.351, *p* = 0.028). In model 2B (HbA1c), SPHS did not show a significant association with HbA1c. Finally, in model 3 (BMI), DE risk was a significant predictor (B = 0.224, *p* = 0.016). Across all models, regression assumptions were met. Residuals showed no evidence of non-normality or heteroscedasticity, and multicollinearity was not a concern, with all tolerance values above 0.5 and all VIF values below 2.

**Table 3:** Unadjusted and adjusted correlations of psychological screening scores and self-perceived health with clinical, anthropometric, biochemical, and self-care variables.

	DDS2S			EPAD-R			SPHS		
	Correlation Coefficient (n)	*p*-Value (CI_95%_)	Adjusted *p*-Value (BH)	Correlation Coefficient (n)	*p*-Value (CI_95%_)	Adjusted *p*-Value (BH)	Correlation Coefficient (n)	*p*-Value (CI_95%_)	Adjusted *p*-Value (BH)
**Age**	−0.181 † (n = 37)	0.280	0.560	0.205 (n = 37)	0.224	0.643	−0.061 (n = 37)	0.718	0.833
**Diabetes duration**	−0.015 ‡ (n = 37)	0.931	0.967	0.235 ‡ (n = 37)	0.162	0.643	0.230 (n = 37)	0.170	0.626
**KIDMED**	0.023 ‡ (n = 37)	0.891	0.967	0.129 ‡ (n = 37)	0.448	0.723	0.103 ‡ (n = 37)	0.546	0.776
**APALQ**	0.011 † (n = 37)	0.947	0.967	0.137 ‡ (n = 37)	0.419	0.723	0.185 ‡ (n = 37)	0.273	0.626
**BMI**	0.195 † (n = 37)	0.247	0.560	0.333 ‡ (n = 37)	0.044 * (0.000/0.599)	0.643	−0.208 ‡ (n = 37)	0.216	0.626
**Percentile**	0.221 † (n = 35)	0.209	0.560	0.255 ‡ (n = 35)	0.145	0.643	−0.178 ‡ (n = 35)	0.313	0.626
**Z-score**	0.260 † (n = 35)	0.131	0.560	0.205 ‡ (n = 35)	0.238	0.643	−0.181 ‡ (n = 35)	0.297	0.626
**Glucose**	0.150 † (n = 30)	0.446	0.637	−0.046 ‡ (n = 30)	0.814	0.942	−0.109 ‡ (n = 30)	0.582	0.776
**Total cholesterol**	0.293 ‡ (n = 28)	0.131	0.560	0.098 ‡ (n = 28)	0.618	0.883	0.121 ‡ (n = 28)	0.540	0.776
**LDL cholesterol**	−0.009 † (n = 24)	0.967	0.967	0.041 ‡ (n = 24)	0.848	0.942	−0.047 ‡ (n = 24)	0.829	0.833
**HDL cholesterol**	0.285 † (n = 24)	0.177	0.560	0.202 ‡ (n = 24)	0.344	0.723	0.321 ‡ (n = 24)	0.127	0.626
**Triglycerides**	0.013 ‡ (n = 30)	0.946	0.967	0.004 ‡ (n = 30)	0.985	0.985	0.041 ‡ (n = 30)	0.833	0.833
**TyG index**	0.160 † (n = 30)	0.415	0.637	0.044 ‡ (n = 30)	0.825	0.942	−0.043 ‡ (n = 30)	0.826	0.833
**TyG-BMI index**	0.225 ‡ (n = 30)	0.240	0.560	0.140 ‡ (n = 30)	0.470	0.723	−0.316 ‡ (n = 30)	0.095	0.626
**Active CGM time**	−0.194 ‡ (n = 36)	0.258	0.560	0.009 ‡ (n = 36)	0.958	0.985	0.155 ‡ (n = 36)	0.504	0.776
**CV**	0.085 ‡ (n = 36)	0.623	0.831	0.067 ‡ (n = 36)	0.697	0.929	0.084 ‡ (n = 36)	0.626	0.783
**TIR**	−0.355 † (n = 36)	0.034 * (−0.612/0.030)	0.340	−0.194 ‡ (n = 36)	0.257	0.643	0.194 ‡ (n = 36)	0.256	0.626
**HbA1c**	0.401 † (n = 37)	0.014 * (0.088/0.642)	0.280	0.234 ‡ (n = 37)	0.163	0.643	−0.330 ‡ (n = 37)	0.046 * (−0.597/0.003)	0.626
**DDS2S**				0.152 ‡ (n = 37)	0.368	0.723	−0.131 ‡ (n = 37)	0.441	0.776
**EPAD-R**	0.152 ‡ (n = 37)	0.368	0.637				−0.204 ‡ (n = 37)	0.225	0.626
**SPHS**	−0.131 ‡ (n = 37)	0.441	0.637	−0.204 ‡ (n = 37)	0.225	0.643			

DDS2S: Spanish translation of the two-item Diabetes Distress Scale; EPAD-R: Encuesta de Problemas Alimentarios en Diabetes–Revisada (the Spanish version of the revised DEPS-R); SPHS: self-perceived health status; CI_95%_: confidence interval; BH: Benjamini–Hochberg correction; KIDMED: Mediterranean Diet Quality Index for children and adolescents; APALQ: Adolescent Physical Literacy Questionnaire; BMI: body mass index; TyG index: triglyceride-glucose index; CV: coefficient of variation; TIR: time in range; HbA1c: glycated hemoglobin; †: Pearson correlation coefficient; ‡: Spearman correlation coefficient; * *p*-value < 0.05 (unadjusted). All *p*-values are two-sided. Pairwise deletion was applied for missing data.

**Table 4:** Unadjusted and adjusted correlations of age and diabetes duration with metabolic and self-care variables.

	Age			Diabetes Duration		
	Correlation Coefficient (n)	*p*-Value (CI_95%_)	Adjusted *p*-Value (BH)	Correlation Coefficient (n)	*p*-Value (CI_95%_)	Adjusted *p*-Value (BH)
**HbA1c**	−0.003 † (n = 37)	0.987	0.987	−0.022 ‡ (n = 37)	0.896	0.909
**CV**	0.354 ‡ (n = 36)	0.034 * (0.019/0.618)	0.292	0.347 ‡ (n = 36)	0.038 * (0.010/0.613)	0.122
**TIR**	−0.064 † (n = 36)	0.710	0.987	−0.045 ‡ (n = 36)	0.793	0.992
**Active CGM time**	−0.068 ‡ (n = 36)	0.692	0.987	−0.051 ‡ (n = 36)	0.766	>1.000
**KIDMED**	−0.120 ‡ (n = 37)	0.478	0.987	−0.073 ‡ (n = 37)	0.668	>1.000
**APALQ**	−0.198 † (n = 37)	0.240	0.640	−0.023 ‡ (n = 37)	0.891	0.956
**BMI**	0.340 † (n = 37)	0.039 * (0.018/0.598)	0.292	0.472 ‡ (n = 37)	0.003 * (0.164/0.695)	0.044 **
**Percentile**	0.035 † (n = 35)	0.845	0.987	0.247 ‡ (n = 35)	0.159	0.424
**Z-score**	0.069 † (n = 35)	0.693	0.987	0.194 ‡ (n = 35)	0.264	0.603
**Glucose**	0.310 † (n = 30)	0.109	0.349	0.474 ‡ (n = 30)	0.011 * (0.112/0.726)	0.044 **
**Total cholesterol**	−0.018 ‡ (n = 28)	0.929	0.987	0.090 ‡ (n = 28)	0.650	>1.000
**LDL cholesterol**	−0.008 † (n = 24)	0.971	0.987	−0.025 ‡ (n = 24)	0.909	0.909
**HDL cholesterol**	−0.141 † (n = 24)	0.512	0.987	0.053 ‡ (n = 24)	0.806	0.992
**Triglycerides**	−0.021 ‡ (n = 30)	0.912	0.987	0.069 ‡ (n = 30)	0.721	>1.000
**TyG index**	0.344 † (n = 30)	0.073	0.292	0.475 ‡ (n = 30)	0.011 * (0.112/0.726)	0.044 **
**TyG-BMI index**	0.352 ‡ (n = 30)	0.061	0.292	0.492 ‡ (n = 30)	0.007 * (0.142/0.733)	0.044 **

CI_95%_: confidence interval; BH: Benjamini–Hochberg correction HbA1c: glycated hemoglobin; CV: coefficient of variation; TIR: time in range; CGM: continuous glucose monitor; KIDMED: Mediterranean Diet Quality Index for children and adolescents; APALQ: Adolescent Physical Literacy Questionnaire; BMI: body mass index; †: Pearson correlation coefficient; ‡: Spearman correlation coefficient; * *p*-value < 0.05 (unadjusted); ** *p*-value (adjusted < 0.05 after Benjamini–Hochberg correction. All *p*-values are two-sided. Pairwise deletion was applied for missing data.

**Table 5:** Multiple linear regression models.

Predictive Variables	Outcome Variables											
	Model 1: TIR			Model 2A: HbA1c			Model 2B: HbA1c			Model 3: BMI		
	B (CI_95%_)	β	*p*-Value	B (CI_95%_)	β	*p*-Value	B (CI_95%_)	β	*p*-Value	B (CI_95%_)	β	*p*-Value
**Sex**	6.026 (−3.539/15.591)	0.215	0.208	−0.411 (−1.017/0.194)	0.297	0.176	−0.503 (−1.119/0.112)	−0.274	0.106	−0.078 (−2.250/2.094)	−0.011	0.942
**Age**	−0.065 (−2.498/2.367)	−0.012	0.957	−0.021 (−0.174/0.133)	−0.059	0.786	−0.091 (−0.247/0.065)	−0.258	0.244	0.125 (−0.415/0.666)	0.092	0.640
**Diabetes duration**	−0.407 (−1.851/1.036)	−0.124	0.569	0.031 (−0.061/0.122)	0.142	0.499	0.069 (−0.027/0.165)	0.176	0.155	0.204 (−0.128/0.535)	0.243	0.219
**DDS2S**	−4.841 (−9.755/0.072)	−0.336	0.053	0.351 (0.040/0.663)	0.153	0.028 *	-	-	-	-	-	-
**EPAD-R**	-	-	-	-	-	-	-	-	-	0.224 (0.044/0.403)	0.389	0.016 *
**SPHS**	-	-	-	-	-	-	−0.463 (−0.983/0.057)	−0.303	0.079	-	-	-

TIR: time in range; HbA1c: glycated hemoglobin; BMI: body mass index; B: unstandardized coefficient; CI_95%_: confidence interval; β: standardized coefficient; DDS2S: Spanish translation of the two-item Diabetes Distress Scale; EPAD-R: Encuesta de Problemas Alimentarios en Diabetes–Revisada (the Spanish version of the revised DEPS-R); SPHS: self-perceived health status; * *p*-value < 0.05; “-“ indicates that the predictor did not apply to the corresponding model.

## 4. Discussion

This study examined the associations between psychological distress, DE risk, SPHS and metabolic control in adolescents with T1DM, highlighting the close interplay between psychological and physiological factors in diabetes management. Psychological distress showed a positive correlation with HbA1c and a negative correlation with TIR. These results align with previous studies which have pointed out that psychological distress and suboptimal metabolic outcomes such as poorer glycemic control may influence each other through complex and potentially bidirectional pathways [31,49,50]. Our findings are consistent with this literature, although the cross-sectional design only allows identification of associations rather than determination of causal direction. Importantly, the association between distress and HbA1c remained significant after adjusting for age, sex and diabetes duration, indicating that this relationship persists beyond demographic factors, while the association with TIR showed a trend toward significance once covariates were considered.

Screening-based DE risk showed a significant positive correlation with BMI, and this relationship remained robust after adjustment for key covariates. Although this association may appear intuitive, it must be interpreted cautiously, since BMI may act both as a contributing factor and a consequence of DE vulnerability, particularly in youth managing strict dietary and insulin regimens [8,12,51]. Higher BMI in adolescents with T1DM has been linked to increased body dissatisfaction and weight-related stigma [12,52], which may trigger maladaptive behaviors such as restrictive eating or intentional insulin omission. Conversely, insulin therapy itself may lead to weight gain and frustration, further reinforcing this cycle [51,53]. These complex interactions point out the need for integrated clinical approaches that jointly address metabolic outcomes and body image concerns. In our sample, DE risk was not significantly associated with any glycemic parameter, including HbA1c, TIR and CV. Although descriptive patterns suggested that adolescents with lower TIR did not necessarily show elevated DE risk, these observations are exploratory and should not be interpreted as evidence of a lack of overlap between DE risk and glycemic variability. Rather, the study did not detect a statistically significant association, and the limited sample size substantially reduces the power to identify potential relationships. Therefore, the absence of significant findings regarding glycemic parameters does not imply that the association does not exist, but that it may not be detectable within the constraints of this study. Larger samples are needed to clarify whether DE risk and glycemic patterns converge during adolescence.

As expected, SPHS was associated with HbA1c [16], perhaps due to a possible tendency among adolescents with T1DM to interpret this biomarker as a key indicator of overall health. However, this finding should be interpreted as exploratory and considered cautiously because the SPHS item showed restricted variability (no participant selected the two lowest response categories), which may weaken the robustness of the correlation analyses. In addition, participants provided their responses after being informed of their HbA1c results during the routine clinical consultation, introducing a potential ordering bias that may have influenced their answers. Future studies should consider assessing SPHS both before and after feedback on metabolic parameters to clarify this relationship [12]. Moreover, this association did not remain significant after adjusting for age, sex and diabetes duration, which may reflect the limited statistical power of our sample rather than the absence of a true relationship between SPHS and HbA1c. It is also important to note that the HbA1c <6.5% threshold used in the descriptive and cross-tabulation analyses is not intended as a universal clinical target. This cut-off was selected because it reflects the criterion applied in the pediatric diabetes clinic where data were collected and aligns with the most recent ISPAD and ADA recommendations [13,19]. In our analyses, HbA1c was treated as a continuous variable, so this descriptive threshold does not influence the inferential results. Age was associated with increased glycemic variability, a pattern that aligns with evidence describing the challenges of adolescence, growing independence and psychological mechanisms such as denial or fatigue regarding the disease [4,5,12,20,21,54]. The positive association between age and BMI may reflect normal pubertal growth and increased muscle mass rather than worsening body composition, especially in males. However, age did not correlate with BMI percentiles or z-scores, which are more robust anthropometric indicators in pediatrics, suggesting that these age-related changes occur within expected growth trajectories [55]. Similarly, diabetes duration correlated with higher CV, BMI, serum glucose, TyG index and TyG-BMI index but not with improved metabolic control. This finding may indicate that a longer disease course does not necessarily translate into better management during adolescence. Instead, it may reflect diabetes-related fatigue or burnout after years of continuous self-care demands [4,20,22]. Nevertheless, glycemic control often improves in emerging adulthood, possibly due to increased maturity, autonomy and coping resources [56]. The associations observed between diabetes duration and TyG-based indices may also reflect early metabolic shifts or subtle changes in insulin sensitivity during adolescence, consistent with emerging evidence suggesting that these markers could offer additional insight into metabolic status in adolescents with T1DM [30]. However, given the limited research available in this population, these interpretations should be considered exploratory.

Lifestyle factors, including adherence to the Mediterranean diet (KIDMED) and physical activity levels (APALQ), did not show significant correlations with metabolic outcomes in our sample. This absence of association may reflect methodological rather than physiological factors, since both instruments were developed for the general adolescent population and are not specifically tailored to individuals with T1DM. For instance, the KIDMED index may not fully capture dietary patterns in youths with diabetes, who intentionally reduce carbohydrate intake to maintain glycemic stability, as these restrictions can be scored as poor adherence to a healthy diet despite reflecting diabetes-specific management strategies [46]. Similarly, the APALQ evaluates overall physical activity but does not account for the dual role of exercise in T1DM, which can be associated with either favorable or unfavorable glycemic patterns depending on insulin dosing, timing and carbohydrate management [57,58]. These limitations underline the need for developing and validating diabetes-specific tools that better capture the complex interplay between lifestyle behaviors and glycemic regulation.

Overall, the psychological, behavioral and metabolic factors examined in this study should not be interpreted in isolation. In adolescents with T1DM, psychological distress, DE risk, SPHS, lifestyle behaviors and age-related developmental changes often coexist and may relate to one another through complex, non-linear patterns. For example, adolescents experiencing psychological distress frequently show less favorable glycemic indicators [31,49,50], and suboptimal glycemic control often coincides with higher adiposity [59], which in turn is associated increased DE vulnerability [8,12,51,52]. These patterns may also intersect with more negative SPHS, particularly when BMI and glycemic outcomes deviate from expected targets [16,17]. Age and diabetes duration can further shape these experiences, influencing adherence, perceived burden and glycemic variability [4,5,20,21,22,54]. Taken together, these interconnected dimensions can jointly influence daily diabetes management, including diet, physical activity patterns and adherence to treatment. Although our cross-sectional design does not allow us to determine directionality, the findings point out the importance of considering these variables within an integrated framework and acknowledge that metabolic outcomes in adolescence emerge from the interplay of multiple psychological, behavioral and physiological influences. Additionally, cross-tabulations provided only preliminary descriptive insight and did not reveal consistent patterns across clinical categories. This lack of clear directionality is expected in small samples, where categorical splits generate imbalanced cells and unstable proportions, limiting the stability of these distributions. For this reason, these tables were interpreted cautiously and considered exploratory visual summaries rather than inferential evidence. More robust insights were obtained from analyses using continuous variables, which avoid artificial dichotomization and provide greater statistical power.

Several limitations should be considered when interpreting these findings, as the study was exploratory in nature. The sample size was relatively small, which restricted the possibility of conducting subgroup analyses by sex, despite known sex differences in psychological distress and DE risk during adolescence. The sample size (n = 37, with smaller Ns for certain biochemical variables) may also have reduced the statistical power to detect small-to-moderate associations. In addition, multivariable models were not performed due to insufficient power, which would compromise model stability and increase the risk of overfitting. These limitations may have influenced our ability to detect subtle associations, underscoring areas where study design could be strengthened. Furthermore, given the number of correlation analyses performed in a relatively small sample, the risk of type I error due to multiple testing is increased, and the reported associations should therefore be interpreted with appropriate caution. Consistent with this, most bivariate correlations did not remain significant after adjusting for multiple comparisons, with the exception of the associations between diabetes duration and BMI, serum glucose, TyG index and TyG-BMI index, which persisted after correction. Notably, these remaining associations were also above the minimum effect size detectable with 80% power (r ≈ 0.42), suggesting that they may represent more robust relationships within the constraints of the sample size.

Additionally, the cross-tabulations should be interpreted with caution due to small and imbalanced cell sizes, which limited the stability of the distributions and reduced their usefulness for inferential purposes. For this reason, these tables were considered exploratory visual summaries rather than robust analytical evidence. Furthermore, several clinically relevant variables that may influence psychological and metabolic outcomes in adolescents with T1DM (such as pubertal stage, insulin-pump or automated insulin-delivery use, insulin dose, recent episodes of severe hypoglycemia or diabetic ketoacidosis, psychiatric diagnoses, psychotropic medication, celiac or thyroid disease, socioeconomic status, parental involvement, and recent weight change) were not systematically collected. The absence of these variables limits the ability to account for potential confounding factors and should be considered when interpreting the reported associations. Future studies should aim to include them to better contextualize psychological and metabolic outcomes. Although the TyG and TyG-BMI indices are increasingly being investigated in adolescents with T1DM, the current evidence base remains limited and their validity in this population is not yet fully established. Accordingly, the interpretation of these indices in our study should be considered exploratory.

The cross-sectional design may also limit the ability to draw causal inferences. In addition, the use of self-report measures may introduce some degree of bias, particularly for subjective constructs such as SPHS or lifestyle behaviors. Alternative designs, including longitudinal follow-up and technology-assisted assessment tools, would likely provide more robust insights into these relationships. Furthermore, questionnaires were completed after the routine follow-up visit, meaning that adolescents were already aware of their glycemic results, which may have influenced their responses to psychological and self-care measures. Although routine pediatric diabetes consultations are often brief and it is challenging to administer questionnaires before the clinical encounter, future studies should consider incorporating this aspect into the protocol to reduce potential response bias related to recent clinical feedback.

Finally, although the DDS2S has been translated into Spanish and used in Spanish research [37,38], no formal psychometric validation exists for Spanish adolescents with type 1 diabetes. The longer DDS (17 items) has been used in Spanish adults with diabetes [60] and has recently undergone formal psychometric validation in Spain [61]. It has also been employed in studies supported by national endocrinology and diabetes societies (SEEN and SED) [62]. This limitation should be considered when interpreting distress estimates in this population. Future research should include larger, diverse cohorts to allow for gender- and age-specific analyses, considering that female adolescents may have higher risk of distress and DE risk [7]. Longitudinal studies are needed to clarify causal pathways and to design interventions addressing both psychological and metabolic health in this population. In addition, developing validated instruments adapted to diabetes would enhance the accuracy and clinical relevance of future findings. Such tools could facilitate the design of targeted interventions addressing both metabolic and psychological needs in adolescents with T1DM. Furthermore, these findings raise questions about how early screening for psychological distress or DE risk could modify metabolic outcomes and patient adherence, suggesting new avenues for research. Clinically, these results encourage closer monitoring of psychological well-being and DE risk in adolescents with T1DM, integrating mental health support alongside metabolic management.

## 5. Conclusions

In conclusion, this study points to the close interplay between psychological and metabolic factors in adolescents with T1DM. Brief screening of psychological distress, DE risk and SPHS appears feasible and showed meaningful cross-sectional associations with metabolic outcomes. These findings point out the potential value of incorporating psychological screening into routine care. Future research with larger and longitudinal cohorts is needed to clarify causal pathways and to determine whether psychological screening can improve clinical outcomes and guide tailored interventions.

## Data Availability

The dataset generated in this study has been deposited in Zenodo under restricted access (DOI: https://doi.org/10.5281/zenodo.17573334). Data are available from the corresponding author upon reasonable request.

## Supplementary Materials

The following supporting information can be downloaded at: https://www.mdpi.com/article/10.3390/nu18172916/s1, Table S1: Crosstabulation between psychological distress and HbA1c categories; Table S2: Crosstabulation between psychological distress and glycemic coefficient of variation categories; Table S3: Crosstabulation between psychological distress and z-score categories; Table S4: Crosstabulation between psychological distress and percentile categories; Table S5: Crosstabulation between psychological distress and total cholesterol categories; Figure S1: Scatterplot of psychological distress and HbA1c; Figure S2: Scatterplot of psychological distress and time in range; Figure S3: Scatterplot of disordered-eating risk and body mass index; Figure S4: Scatterplot of self-perceived health status and HbA1c; Figure S5: Scatterplot of age and glycemic coefficient of variation; Figure S6: Scatterplot of diabetes duration and glycemic coefficient of variation; Figure S7: Scatterplot of diabetes duration and serum glucose; Figure S8: Scatterplot of diabetes duration and triglyceride-glucose index.

## Abbreviations

The following abbreviations are used in this manuscript:

T1DM	Type 1 diabetes mellitus
DE	Disordered-eating
BMI	Body mass index
CGM	Continuous glucose monitoring
SPHS	Self-perceived health status
DDS2S	Diabetes Distress Scale, two-item short form
DEPS-R	Diabetes Eating Problem Survey–Revised
EPAD-R	Encuesta de Problemas Alimentarios en Diabetes–Revisada (the Spanish version of the DEPS-R)
CV	Coefficient of variation
TIR	Time in range
HbA1c	Glycated hemoglobin
LDL	Low-density lipoprotein cholesterol
HDL	High-density lipoprotein cholesterol
TyG	Triglyceride–glucose index
TyG-BMI	Triglyceride–glucose index adjusted for body mass index
ISAK	International Society for the Advancement of Kinanthropometry
WHO	World Health Organization
CDC	U.S. Centers for Disease Control and Prevention
ISPAD	International Society for Pediatric and Adolescent Diabetes
ADA	American Diabetes Association
STROBE-nut	Strengthening the Reporting of Observational Studies in Epidemiology–Nutrition extension
APALQ	Adolescent Physical Literacy Questionnaire
KIDMED	Mediterranean Diet Quality Index for children and adolescents
