# Supplementary material for: Associations of Diabetes-Related Distress, Disordered-Eating Risk, and Self-Perceived Health with Glycemic Outcomes in Adolescents with Type 1 Diabetes: An Exploratory Cross-Sectional Study
Source: Nutrients. 2026 Sep 5;18(17):2916. doi: 10.3390/nu18172916 (PMC13567549; doi:10.3390/nu18172916)
Supplement: Supplementary file 1 [file nutrients-18-02916-s001.zip › Supplementary material - Cross-tabulations.pdf]

Supplementary Table S1. Cross-tabulation between psychological distress and HbA1c categories.

DDS2S categories	HbA1c ≤ 6.5 % (controlled)	HbA1c > 6.5 % (not controlled)	Total
≥ 3 points (presence of distress)	6 (66.7%)	13 (46.4%)	19 (51.4%)
< 3 points (absence of distress)	3 (33.3%)	15 (53.6%)	18 (48.6%)
Total	9 (100%)	28 (100%)	37 (100%)

DDS2S: Spanish translation of the 2-item Diabetes Distress Scale; HbA1c: Glycated hemoglobin.

Supplementary Table S2. Cross-tabulation between psychological distress and glycemic coefficient of variation categories.

DDS2S categories	CV ≤ 36 % (controlled)	CV > 36 % (not controlled)	Total
≥ 3 points (presence of distress)	4 (66.7%)	14 (46.7%)	18 (50%)
< 3 points (absence of distress)	2 (33.3%)	16 (53.3%)	18 (50%)
Total	6 (100%)	30 (100%)	37 (100%)

DDS2S: Spanish translation of the 2-item Diabetes Distress Scale; CV: Coefficient of variation.

Supplementary Table S3. Cross-tabulation between psychological distress and z-score categories.

DDS2S categories	Z-score < 1 (normal weight)	Z-score ≥ 1 (overweight or obesity)	Total
≥ 3 points (presence of distress)	13 (52.0%)	4 (40%)	17 (48.6%)
< 3 points (absence of distress)	12 (48%)	6 (60%)	18 (51.4%)
Total	25 (100%)	10 (100%)	35 (100%)

DDS2S: Spanish translation of the 2-item Diabetes Distress Scale.

Supplementary Table S4. Cross-tabulation between psychological distress and percentile categories.

DDS2S categories	Percentile < 85 (normal weight)	Percentile ≥ 85 (overweight or obesity)	Total
≥ 3 points (presence of distress)	13 (52.0%)	4 (40%)	17 (48.6%)
< 3 points (absence of distress)	12 (48%)	6 (60%)	18 (51.4%)
Total	26 (100%)	10 (100%)	35 (100%)

DDS2S: Spanish translation of the 2-item Diabetes Distress Scale.

Supplementary Table S5. Cross-tabulation between psychological distress and total cholesterol categories.

DDS2S categories	C-total < 170mg/dL (controlled)	C-total ≥ 170mg/dL (uncontrolled)	Total
≥ 3 points (presence of distress)	9 (60%)	4 (30.8%)	13 (46.4%)
< 3 points (absence of distress)	6 (40%)	9 (69.2%)	15 (53.6%)
Total	15 (100%)	13 (100%)	28 (100%)

DDS2S: Spanish translation of the 2-item Diabetes Distress Scale; C-total: Total cholesterol.
