# Supplementary material for: Associations of Diabetes-Related Distress, Disordered-Eating Risk, and Self-Perceived Health with Glycemic Outcomes in Adolescents with Type 1 Diabetes: An Exploratory Cross-Sectional Study
Source: Nutrients. 2026 Sep 5;18(17):2916. doi: 10.3390/nu18172916 (PMC13567549; doi:10.3390/nu18172916)
Supplement: Supplementary file 1 [file nutrients-18-02916-s001.zip › Supplementary material - Scatterplots.pdf]

Supplementary Figure S1. Scatterplot of psychological distress and HbA1c.

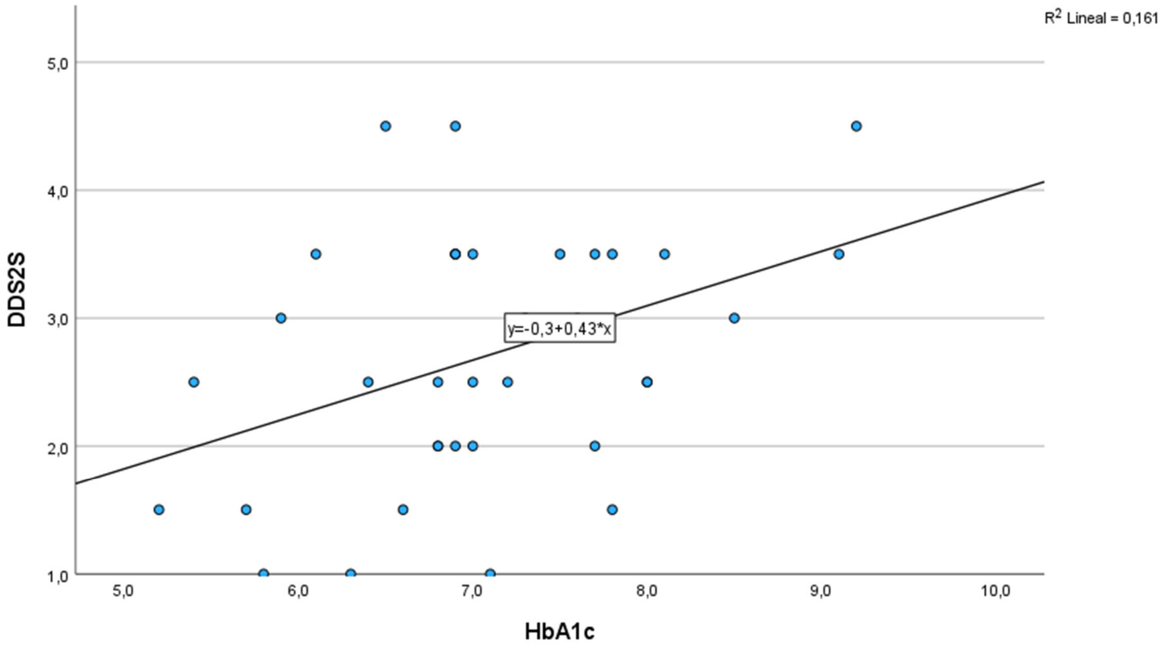

Scatterplot illustrating the distribution and linear trend between diabetes distress (DDS2S; Spanish translation of the 2-item Diabetes Distress Scale) and HbA1c levels in adolescents with type 1 diabetes mellitus.

Supplementary Figure S2. Scatterplot of psychological distress and time in range.

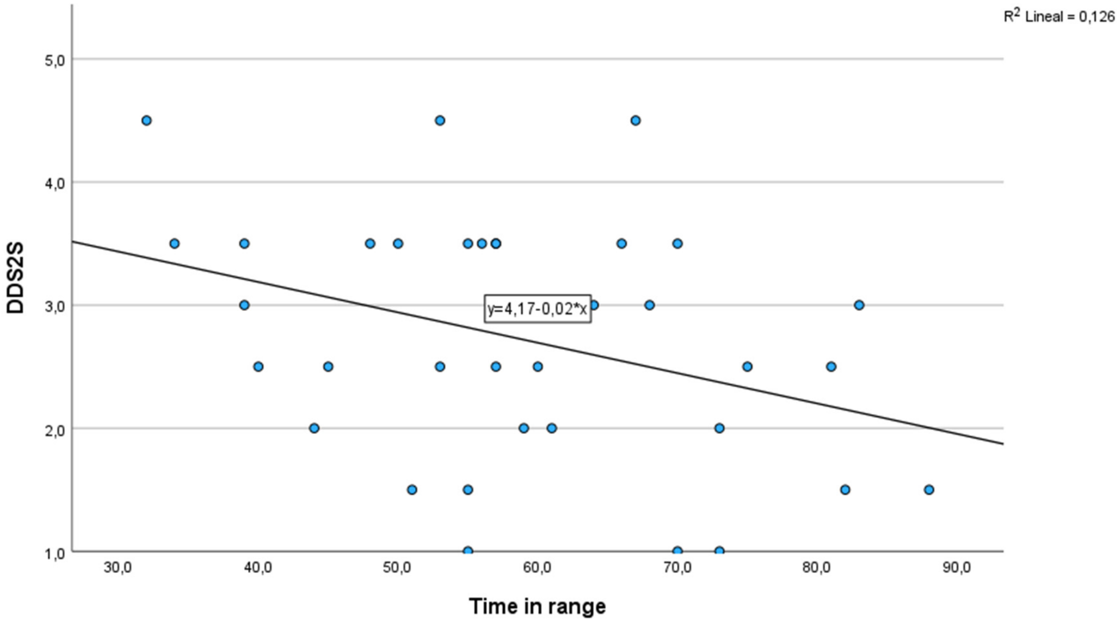

Scatterplot illustrating the distribution and linear trend between diabetes distress (DDS2S; Spanish translation of the 2-item Diabetes Distress Scale) and time in range in adolescents with type 1 diabetes mellitus.

Supplementary Figure S3. Scatterplot of disordered-eating risk and body mass index.

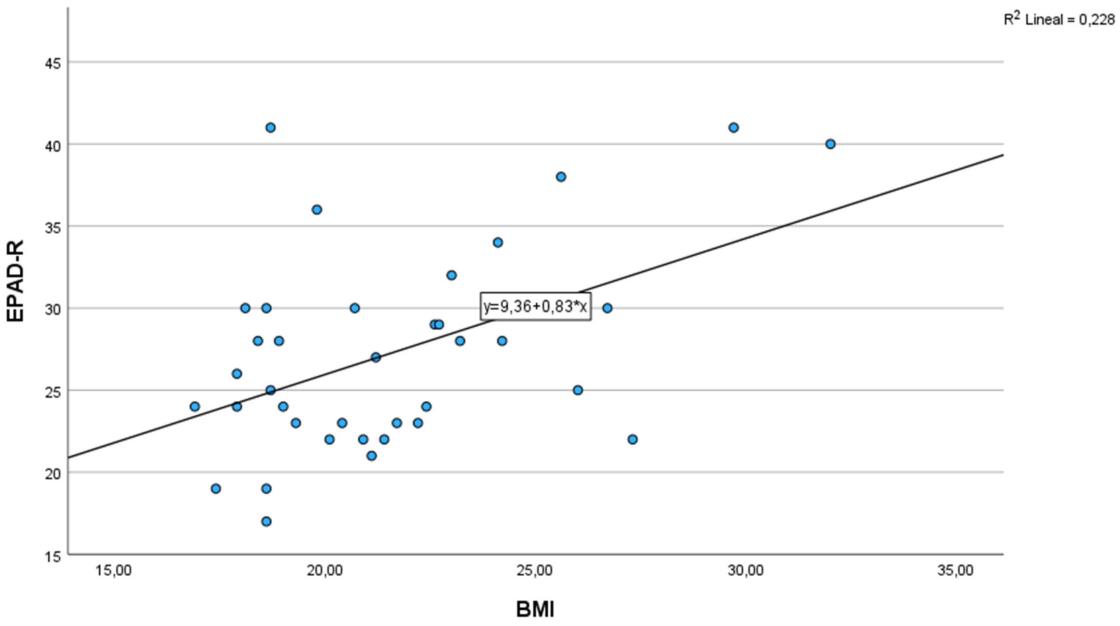

Scatterplot illustrating the distribution and linear trend between disordered-eating risk (EPAD-R) and body mass index (BMI) in adolescents with type 1 diabetes mellitus.

Supplementary Figure S4. Scatterplot of self-perceived health status and HbA1c.

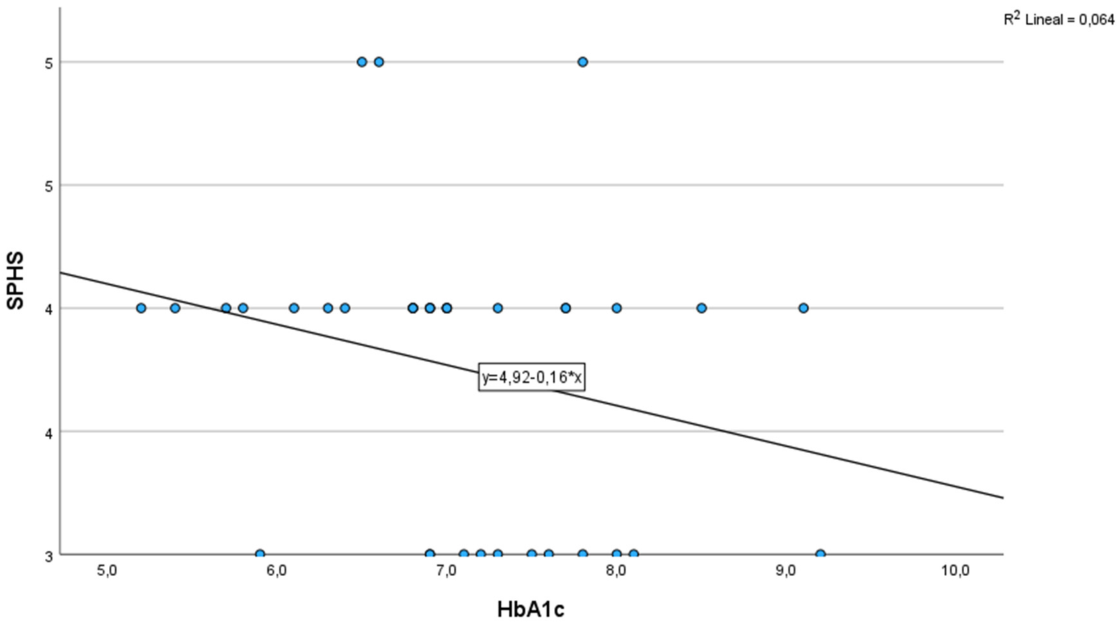

Scatterplot illustrating the distribution and linear trend between self-perceived health status (SPHS) and HbA1c levels in adolescents with type 1 diabetes mellitus.

Supplementary Figure S5. Scatterplot of age and glycemic coefficient of variation.

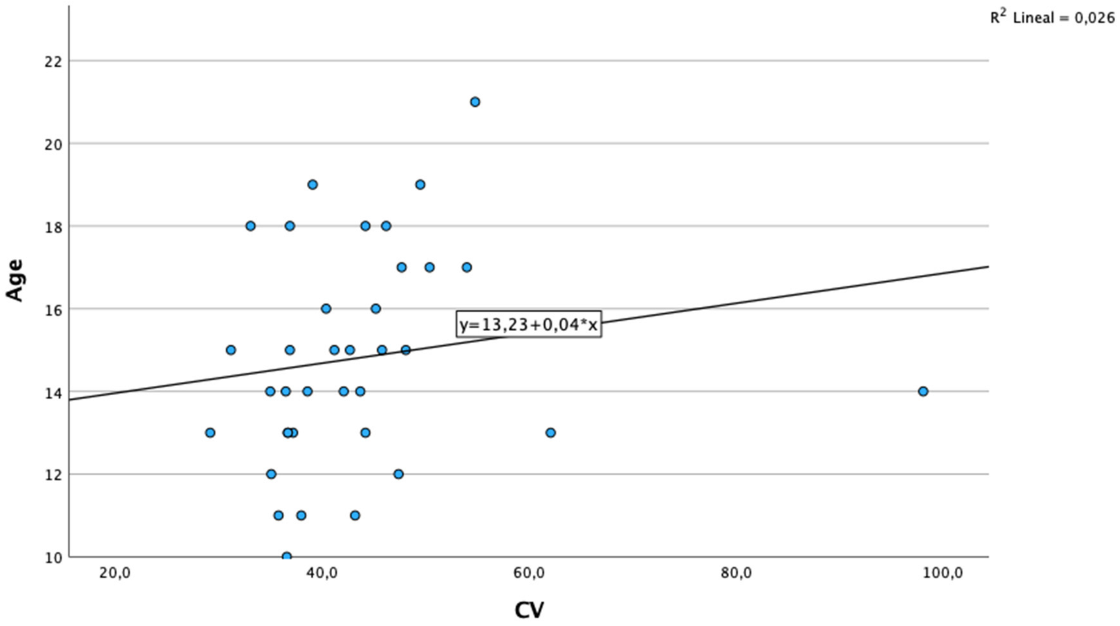

Scatterplot illustrating the distribution and linear trend between age and glycemic coefficient of variation (CV) in adolescents with type 1 diabetes mellitus.

Supplementary Figure S6. Scatterplot of diabetes duration and glycemic coefficient of variation.

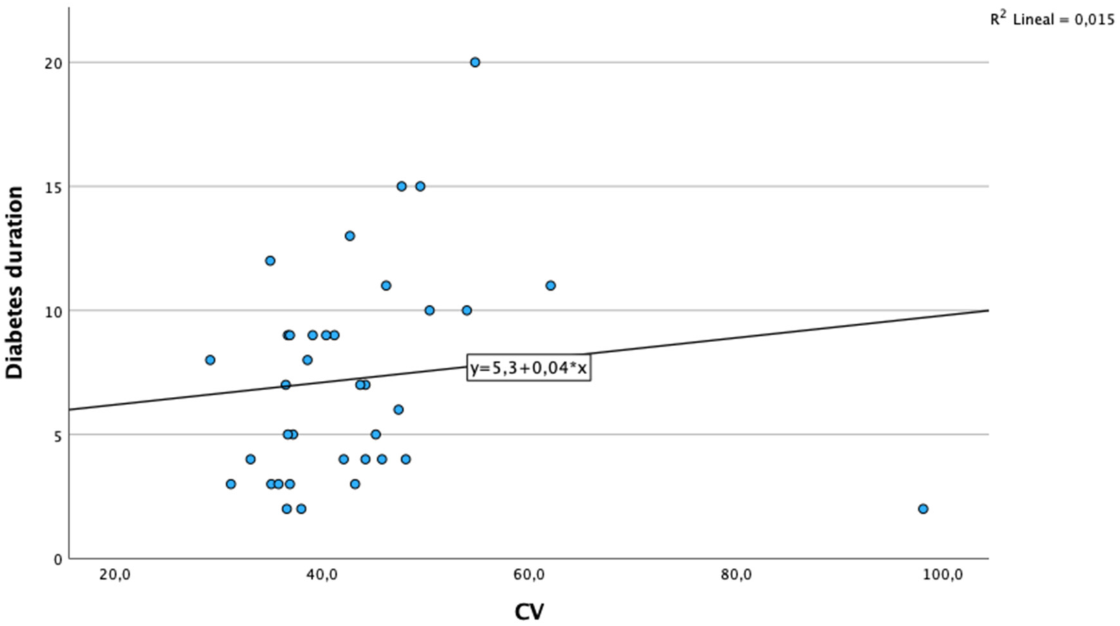

Scatterplot illustrating the distribution and linear trend between diabetes duration and glycemic coefficient of variation (CV) in adolescents with type 1 diabetes mellitus.

Supplementary Figure S7. Scatterplot of diabetes duration and serum glucose.

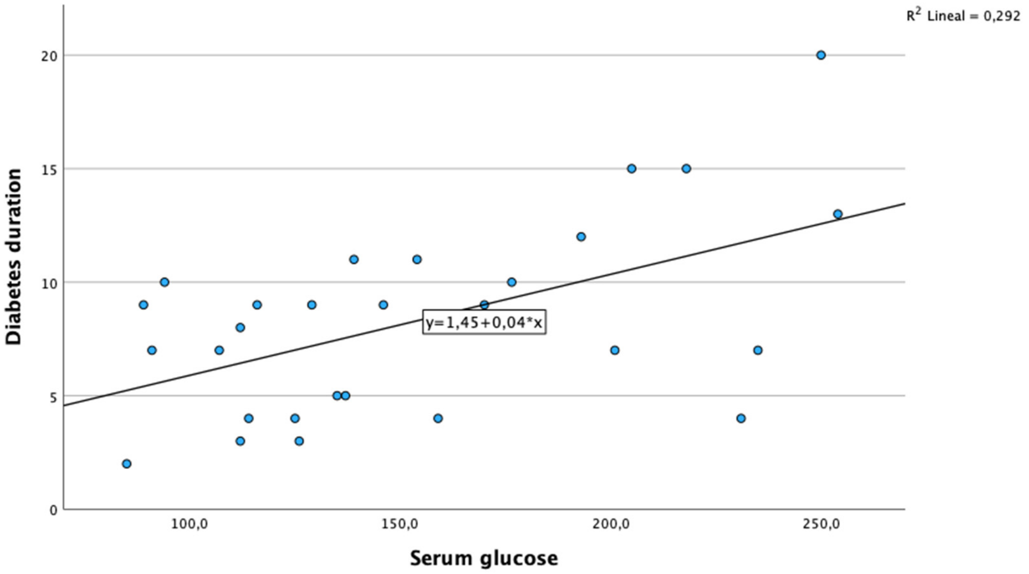

Scatterplot illustrating the distribution and linear trend between diabetes duration and serum glucose levels.

Supplementary Figure S8. Scatterplot of diabetes duration and triglyceride-glucose index.

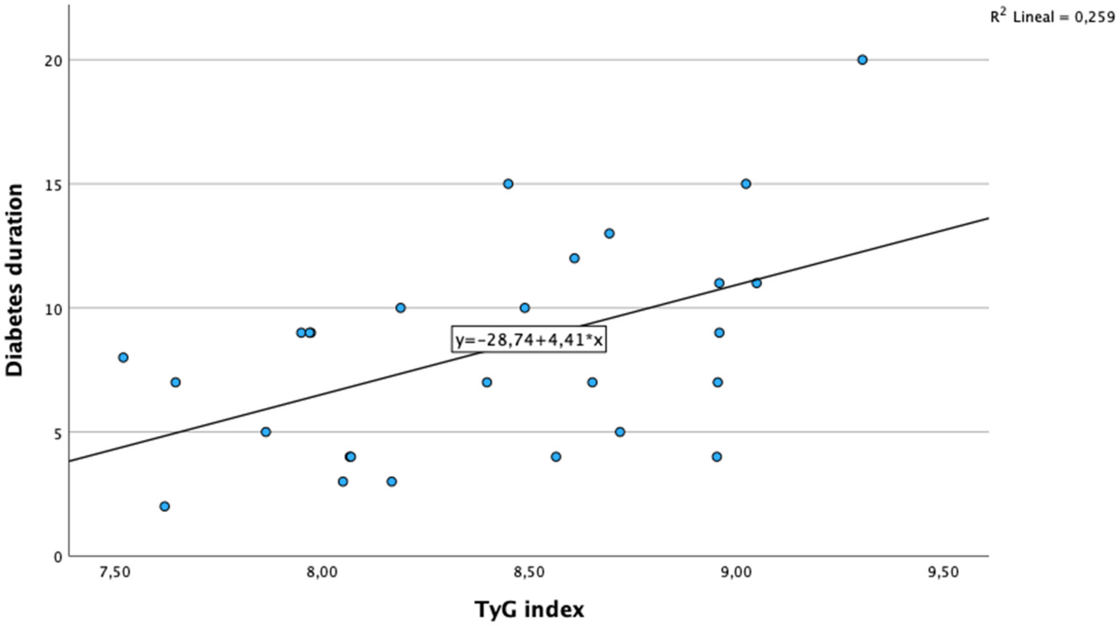

Scatterplot illustrating the distribution and linear trend between diabetes duration and the triglyceride-glucose (TyG) index.
